# Transcriptomic Mapping of Non-Small Cell Lung Cancer *K-RAS* p.G12C Mutated Tumors: Identification of Surfaceome Targets and Immunologic Correlates

**DOI:** 10.3389/fimmu.2021.786069

**Published:** 2022-02-01

**Authors:** Ana Alcaraz-Sanabria, Esther Cabañas Morafraile, Gonzalo Fernández-Hinojal, Guillermo Velasco, Pedro Pérez-Segura, Atanasio Pandiella, Balázs Győrffy, Alberto Ocaña

**Affiliations:** ^1^ Translational Oncology Laboratory, Centro Regional de Investigaciones Biomédicas, Castilla-La Mancha University (CRIB-UCLM), Albacete, Spain; ^2^ Experimental Therapeutics Unit, Medical Oncology Department, Hospital Clínico Universitario San Carlos (HCSC), Instituto de Investigación Sanitaria San Carlos (IdISSC) and Centro de Investigación Biomédica en Red en Oncología Centro (CIBERONC), Madrid, Spain; ^3^ Center for Biological Research Margarita Salas (CIB-CSIC), Spanish National Research Council, Madrid, Spain; ^4^ Department of Biochemistry and Molecular Biology, School of Biology, Complutense University, Madrid, Spain; ^5^ Instituto de Biología Molecular y Celular del Cáncer (IBMCC-CSIC), Instituto de Investigación Biomédica Salamanca (IBSAL) and Centro de Investigación Biomédica en Red en Oncología (CIBERONC), Salamanca, Spain; ^6^ Department of Bioinformatics and 2^nd^ Department of Paediatrics, Semmelweis University, Budapest, Hungary; ^7^ Research Centre for Natural Sciences (TTK) Lendület Cancer Biomarker Research Group, Institute of Enzymology, Budapest, Hungary

**Keywords:** *K-RAS*, lung adenocarcinoma, surfaceome, genomic signature, *CLDN10* and *TMPRSS6*

## Abstract

Targeting K-RAS-mutant non-small cell lung cancer (NSCLC) with novel inhibitors has shown promising results with the recent approval of sotorasib in this indication. However, progression to this agent is expected, as it has previously been observed with other inhibitors. Recently, new immune therapeutics, including vectorized compounds with antibodies or modulators of the host immune response, have demonstrated clinical activity. By interrogating massive datasets, including TCGA, we identified genes that code for surface membrane proteins that are selectively expressed in K-RAS mutated NSCLC and that could be used to vectorize novel therapies. Two genes, *CLDN10* and *TMPRSS6*, were selected for their clear differentiation. In addition, we discovered immunologic correlates of outcome that were clearly de-regulated in this particular tumor type and we matched them with immune cell populations. In conclusion, our article describes membrane proteins and immunologic correlates that could be used to better select and optimize current therapies.

**Graphical Abstract:**
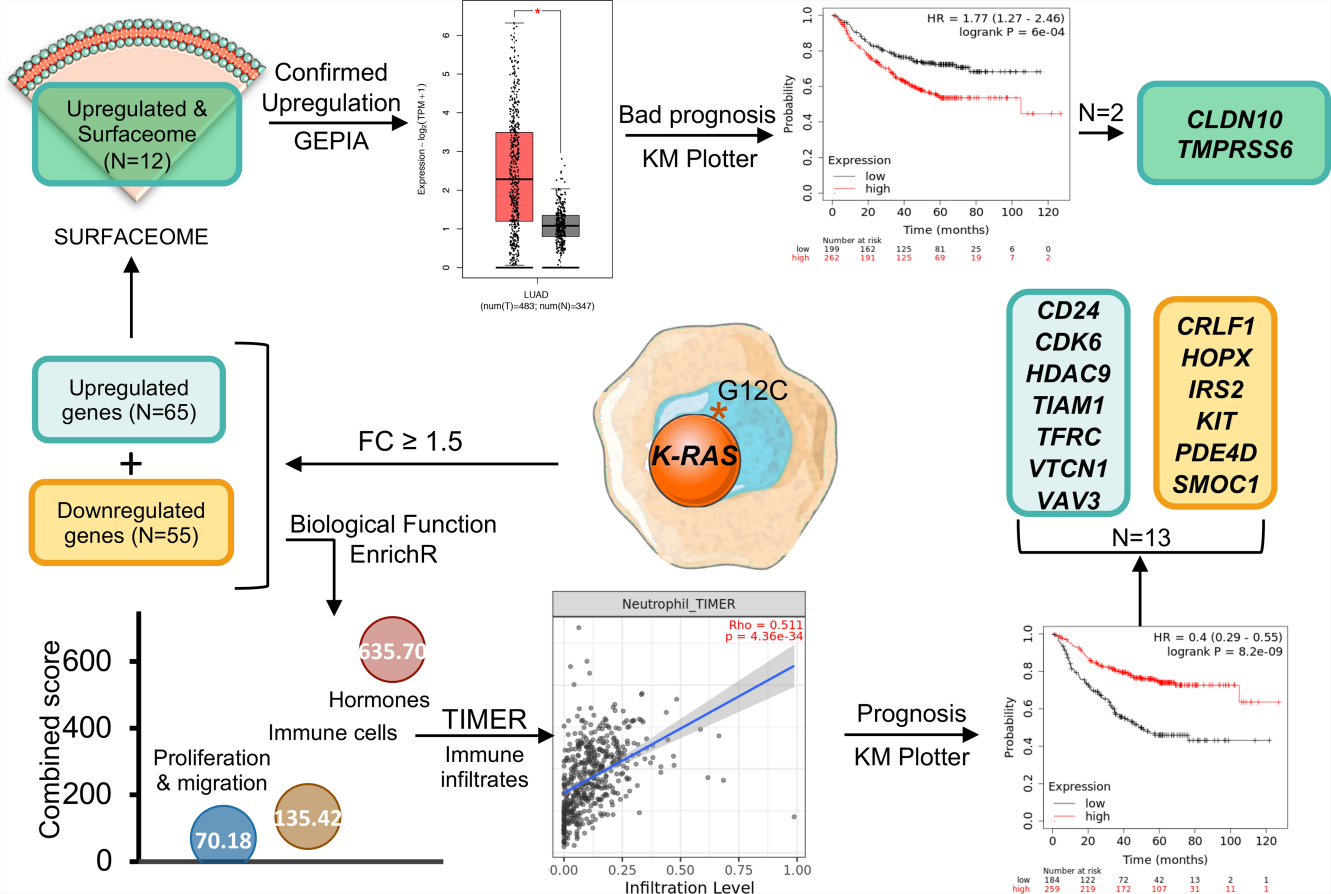


## Introduction

Identification of the key molecular alterations that drive oncogenesis is one of the main objectives in current cancer research (1). These alterations are essential to regulate tumor generation and progression but, at the same time, generate vulnerabilities that can be therapeutically exploited. Thus, in the last few decades much effort has been put on the development of anticancer treatments selectively targeting gene products that become dysregulated as a consequence of those oncogenic alterations. This strategy, combined with the generalization of the use of molecular diagnosis, has led to the application of more individualized and selective treatments which has conducted to a very significantly improvement in patient survival in several types of cancer (2, 3). Two examples of this approach have been the development and utilization of neutralizing antibodies and small molecule inhibitors against the HER2 tyrosine kinase receptor in breast and gastric tumors, and the development of inhibitors against the BRAF V600E mutation in melanoma (4). Recently we have seen a novel example of these type of selective therapies with the development of agents against the K-RAS pG12C mutation in non-small cell lung cancer (NSCLC). K-RAS is a small G protein, and one of the most frequently mutated genes in cancer. Most mutations on this gene occur at the Glycine 12 (G12) residue, which renders K-RAS constitutively active by favouring its GTP-bound active form. Activated GTP-bound K-RAS stimulates many different intracellular signalling pathways such as the RAF1/MEK/ERK and PI3K cascades that are directly involved in the regulation of cell proliferation, motility etc (5). The paramount importance of K-RAS mutations in cancer has led to a prolonged effort to develop strategies to target this protein that however was considered to be undruggable due to its structural characteristics. However, the presence of a thiol group at Cys 12 of K-RAS G12C offered an opportunity to develop inhibitors against this specific mutant, which is present with a notable frequency in certain cancer types and specifically in NSCLC (6). Following this strategy, a new family of compounds aimed at targeting K-RAS G12C has been developed. One of these compounds (AMG-510 or Sotorasib) acts by holding K-RAS G12C in its GDP-bound inactive form and has recently been granted accelerated approval for the treatment of *K-RAS G12C-*mutated (7) locally advanced or metastatic NSCLC using a specific companion diagnostic (8). Likewise, other compounds acting on the same target are currently in clinical development (9).

Targeting oncogenic membrane kinase proteins with antibodies or small molecule signalling mediators binding to the kinase pocket has shown to induce clinical benefit. However, this effect is short lived, and resistance frequently appears within a short period of time (10). Several mechanisms of resistance have been described, and most overcome the activity inhibited by the compound through the activation of alternative pathways, acquisition of secondary mutations at the kinase pocket, or the presence of gene amplifications or alternative splicing, among others (10). Amid these, the development of secondary mutations is a key mechanism described for many kinase inhibitors like those acting on the EGF receptor in lung cancer (11). Similar findings involving secondary mutations have been reported recently with inhibitors against K-RAS G12C (12). In this context, the identification of smart combinations – including the association with novel immunotherapies – is a main goal in this particular clinical scenario.

An interesting approach to overcome resistance due to specific mutations is reducing the abundance of the mutated protein by promoting its selective degradation (13). One strategy to follow this approach, and that is currently intensively investigated, is the utilization of Proteolysis Targeting Chimeras (PROTAC) compounds which through their binding with specific E3 ligases, induce the ubiquitination and subsequent degradation of the target protein (13). This strategy has proved to be effective in preclinical models where it has shown to overcome the resistance associated with several mutations, including those present at the androgen receptor (14), thus warranting further investigation to substantiate its clinical development. Likewise, PROTACs have been recently synthetized with this aim of overcoming resistance against K-RAS G12C inhibitors (15). However, these compounds, like many others agents against pan-essential genes, suffer from potential toxicity, as they act against non-transformed tissues with a potent off-target effect, limiting their clinical development (4). One of the ways to avoid this problem is the identification of proteins that are exclusively present in the tumor surface and that could therefore be used as selective targets for the vectorization of novel compounds (3). This has been the case with guided chemotherapeutics like antibody drug conjugates (ADCs), that have shown to improve survival in several indications with manageable toxicity profiles (16). In this context, an important requisite is the identification of proteins specifically expressed at the membrane of tumor cells.

Immune therapy has gained momentum in NSCLC with several immune check point inhibitors (ICIs) demonstrating clinical efficacy including those targeting PD (L)1, CTLA-4, and some others, as anti- TIGIT or LAG-3 therapies, that have shown early signs of activity (17). Modulation of the immune response in tumors with specific driver mutations can pave the way for potential combinations with ICIs or other immune therapies. For instance, tumors holding EGFR mutations develop an immune suppressive microenvironment which is probably the reason why the combination of inhibitors of EGFR and PD (L)1 has demonstrated limited efficacy (18). In contrast, the combination of the K-RAS G12C inhibitor adagrasib with ICIs induced a remarkable antitumor activity warranting further exploration in preclinical and clinical studies (19). In this scenario, the identification of specific immune regulatory mechanisms that could operate in *K-RAS* G12C mutated tumors would be of great interest as it would help to design novel therapeutic strategies based on the combination of drugs that could potentially overcome the resistance to drugs targeting this mutation, and or reactivate the immune response in these tumors.

In our study, we analysed deregulated genes expressed in NSCLC harbouring mutations at K-RAS pG12c. We applied our approach to reach two goals: the identification of specific membrane proteins that could be the target for the design of antibody guided compounds; and exploring immune related deregulated pathways present in this tumor type that could be used to design novel drug combinations or used as surrogate immune signatures.

## Results

### Landscape of Genomic Variants of *K-RAS* Mutated Tumors and Ongoing Therapeutics

**Figure 1:**
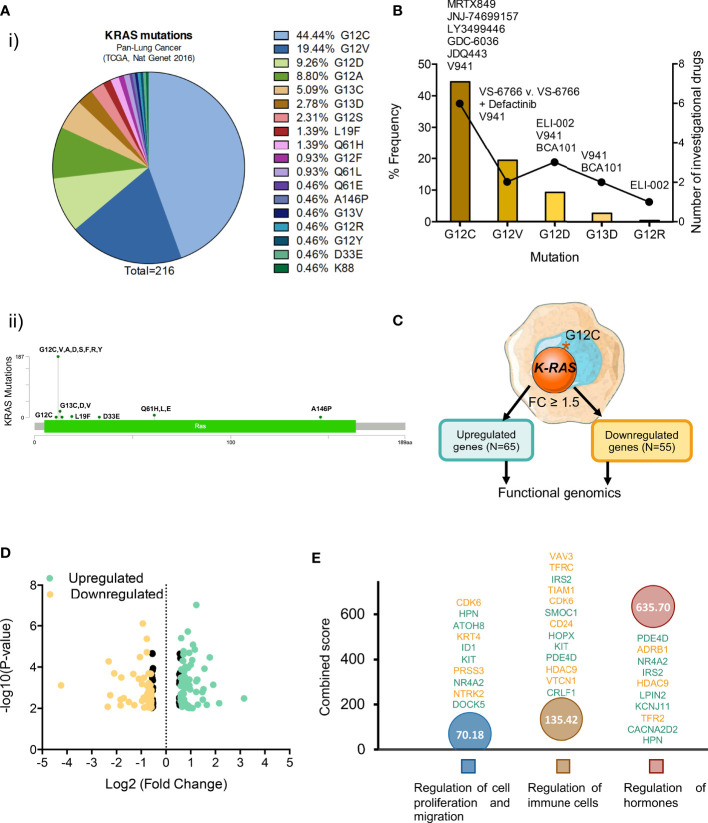
Most common K-RAS mutations in patients with NSCLC and investigational drugs against these mutations. **(A) (i)** Frequency of K-RAS mutations in NSCLC patient population **(ii)** Distribution of mutations identified in patients with lung adenocarcinoma along K-RAS gene. **(B)** Dot chart showing the number of investigational drugs and bar graph representing the percentage frequency of most common K-RAS mutations **(C)** Flow chart of the process followed to select upregulated (N=65) and downregulated (N=55) differentially expressed genes. **(D)** Volcano plot of statistic significant deregulated genes highlighted in color (yellow for down and green for upregulated) those with fold change equal or higher than 1.5. **(E)** Graph showing the biological processes of the selected 120 genes with higher combine score.

To get insight into potential therapeutic opportunities in Lung Squamous Cell Carcinoma (LSCC) we first explored the *K-RAS* mutational landscape in this molecular tumor subtype. Less than 1.5% of patients expressed this mutation (TCGA, PanCancer Atlas database: 1.49%), while in the Adenocarcinoma subtype (LUAD), mutations at *K-RAS* were observed in around 35% of patients (37.91% and 29.13% in MSKK 2020 and TCGA Firehose Legacy databases, respectively) (**Supplementary Figure 1**). The most frequent mutation described is the one present at the position 12 that changes a Glycine for different aminoacids, such as G12C observed in 44.44% of patients, followed by G12V in 19.44%, G12D in 9.26% and G12A in 8.80% of patients. In the same position, there are also other mutations less frequently observed: G12S (2.31%), G12F (0.93%), G12R (0.46%), G12Y (0.46%). In summary, the frequent mutations observed at the G12 residue (or the closest ones, like G13 further support the notion that this region is a cancer hotspot in this tumor sybtype (**Figure 1A**).

When exploring therapeutic options against the described molecular alterations, we observed that only one drug, sotorasib (AMG 510) has been approved for the treatment of *K-RAS* G12C mutated NSCLC, with another one, adagrasib in its late clinical development stage. Moreover, although the possibility of targeting other K-RAS mutants including G12C, G12D, G12V, G13D, and G12R is being evaluated (**Figure 1B**) most drugs currently under development have been designed against the K-RAS G12C mutation (**Figures 1B, C** and **Supplementary Table 1**).

### Transcriptomic Mapping of K-RAS pG12c Mutated Tumors in NSCLC

Given the fact that K-RAS pG12C mutation is most frequently observed in NSCLC and there is already an approved drug for this indication, we explored the transcriptomic profile of these tumors in order to identify immune genomic alterations with potential for clinical translation. For this purpose, we interrogated public datasets to identify genes that were up- or downregulated in lung tumors harbouring this mutation (**Figure 1C**). First, we focused on genes with a fold changed differential expression > 1.5 (**Figure 1D**), what let us to identify 65 upregulated and 55 downregulated transcripts (**Figures 1C, D**). Next, we explored the biological functions of these deregulated genes using the EnrichR platform and found that they were mainly associated with the regulation of cell proliferation and migration, immune cells and hormone signalling (**Figure 1E**). A full description of the genes that are deregulated and the associated biological functions is shown in **Supplementary Table 2**.

**Figure 2:**
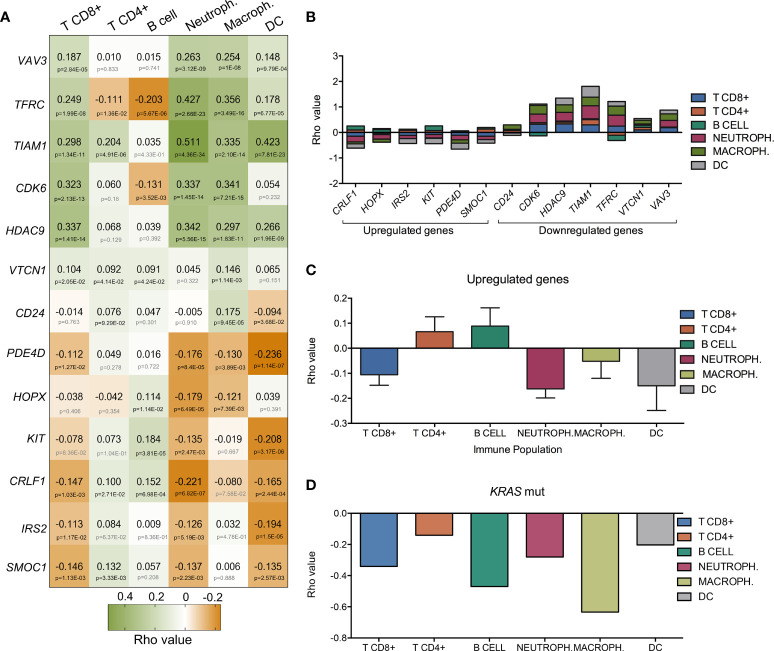
Association of the expression of the thirteen selected genes with immune infiltrates in LUAD. **(A)** Heatmap that represents the correlation (green for positive Rho value and orange for negative one) between gene-expression and the level of tumor immune infiltrates (CD8+ T cells, CD4+ T cells, B-cells, macrophages, neutrophils and dendritic cells). Bellow Rho value, p-value is shown (in grey if no significant and in black if significant). **(B)** Bar graph showing the accumulative correlation to all immune populations named above with the expression of up and downregulated genes**. (C)** Rho values indicating the correlation between expression of upregulated genes (*CRLF1, HOPX, IRS2, KIT, PDE4D*, and *SMOC1*) and immune populations. **(D)** Correlation between *K-RAS* mutated gene and immune infiltrates.

Since immune cell regulation is one of the biological functions with higher combined score, we decided to evaluate the correlation between the expression of these genes and the presence of immune infiltrates (**Figure 2A**). Surprisingly, downregulated genes involved in these functions had a positive correlation with immune infiltrates while upregulated genes displayed a negative association (**Figure 2B**). Thus, high expression of *CLRF1, HOPX, IRS2, KIT, PDE4D*, and *SMOC1*, was associated with a reduction of neutrophils, macrophages, CD8+ T lymphocytes and dendritic cells (**Supplementary Figure 2**) with the only exception of an increase of B and CD4+ T cells, as shown in **Figure 2C**. Likewise, downregulation of *CD24, CDK6, HDAC9, TIAM1, TRFC, VTCN1*, and *VAV3*, correlated with a reduction of the presence of the same immune populations (**Supplementary Figure 3**). We next wondered if LUAD cancers with mutated *K-RAS* had a positive or negative correlation with immune infiltrates when compared with their wild type counterparts. As shown in **Figure 2D**, and in line with the above observations, *K-RAS* mutated LUAD tumors exhibited a negative correlation with all populations of immune infiltrates. In this context, if we compare the correlation of the immune infiltrates associated with mutated K-RAS (**Figure 2D**) with the one that associates with the upregulation of the selected genes (**Figure 2C**), we observe similar levels of correlation with CD8+ T cells, neutrophils and DC and an increase in the CD4+ T cells, B cells and macrophages. This fact indicates that upregulation of those genes may have a role in the relation of the CD4+ T cells, B cells and macrophages populations within the tumor.

### Expression Changes Associated to Mutated K-RAS Predicts Favorable Outcome in Lung Cancer

**Figure 3:**
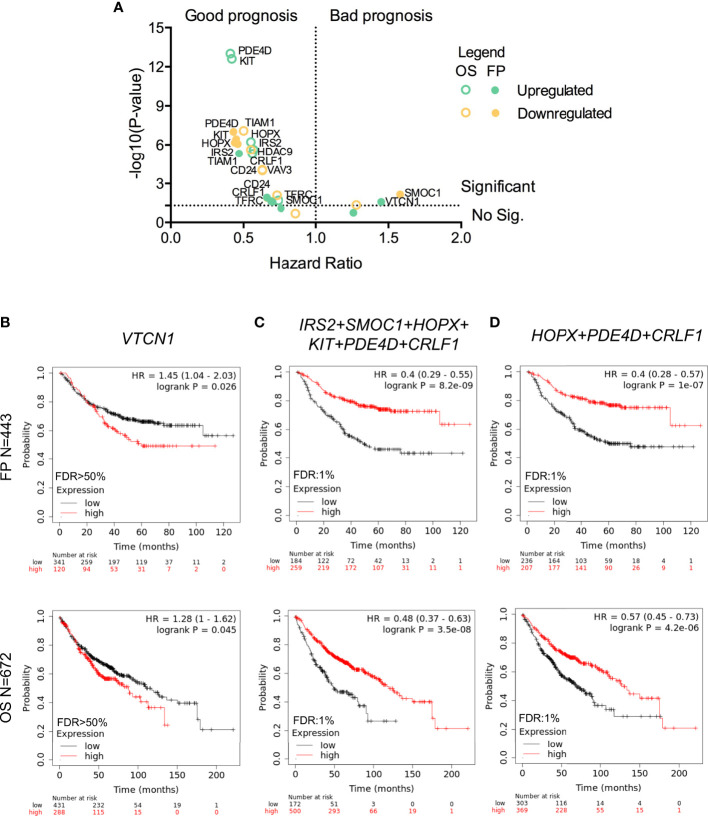
Association between gene expression and LUAD survival. **(A)** Dot plot indicating the – log (P-value) and hazard ratio from Kaplan-Meier of the individual expression of the thirteen selected genes (green for upregulated and yellow for downregulated ones) to FP (filled dots) or OS (empty dots). Kaplan-Meier survival plots showing the association between the single (*VTCN1* in **B**) or combined gene expression levels (*IRS2, SMOC1, HOPX, KIT, PDE4D*, and *CRLF1* in **(C)**; and *HOPX, PDE4D*, and *CRLF1* in **D**) with FP (above) and OS (below) for LUAD.

The KM plotter tool was used to explore the prognostic value of the 13 genes identified in the previous analysis that had a fold change higher than 1.5 and the highest combined score according to their biological functions. The expression of most of those genes was associated with good prognosis when using first progression (FP) and overall survival (OS) as endpoints with the only exception of *SMOC1* and *VTCN1* whose expression conferred detrimental FP (**Figure 3A**). VTCN1 negatively regulates T-cell-mediated immune responses by inhibiting T-cell activation, proliferation, cytokine production and cytotoxicity (20, 21). In line with the identification of VTCN1 as a promising target for cancer treatment, its expression was associated with a shorter FP (HR:1.45, 95% CI 1.04–2.03, log rank p = 0.026) and a poor OS (HR:1.28, 95% CI 1–1.62, log rank p = 0.045) (**Figure 3B**).

For upregulated genes, we observed that almost all of them correlated with better prognosis. When we used the expression of *IRS2, SMOC1, HOPX, KIT, PDE4D*, and *CRLF1* as a signature, we obtained a better association with favorable outcome for FP (HR:0.4, 95% CI 0.29–0.55, log rank p = 8.2 × 10^−9^) and OS (HR:0.48, 95% CI 0.37–0.63, log rank p = 3.5 × 10^−8^) (**Figure 3C**). This result was reproduced when the combined expression of *HOPX, PDED4* and *CRLF1* was analyzed. Therefore, these genes showed a similar good prognostic association when FP (HR:0.4, 95% CI 0.28–0.57, log rank p = 1 × 10^−7^) and OS (HR:0.57, 95% CI 0.45–0.73, log rank p = 4.2 × 10^−6^) were considered (**Figure 3D**).

### Surfaceome Transcriptomic Mapping of K-RAS

**Figure 4:**
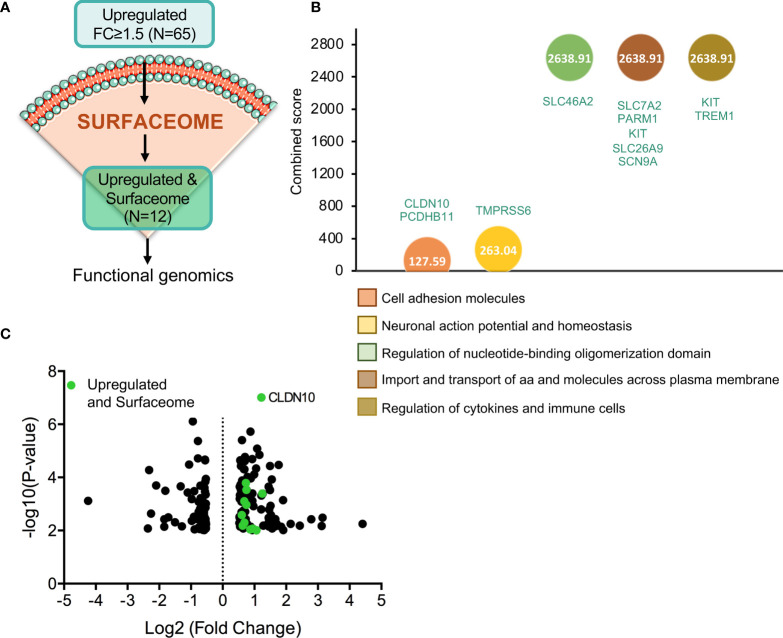
Gene identification as cell-surface target candidates. **(A)** Flow chart of the process followed in order to identify those upregulated genes with fold change equal or higher than 1.5 (N=65) that codify for cell-surface proteins (N=12). **(B)** Identification of the biological processes with higher combine score in which the 12 selected genes are involved. **(C)** Volcano plot of statistic significant deregulated genes highlighted in green those that belong to surfaceome.

As mentioned in the introduction, one of the strategies to develop more selective anticancer therapies is the identification of potential targets that are present in the plasma membrane of cancer cells. Hence, we analysed whether any of the 65 upregulated genes described before coded for plasma membrane proteins, identifying 12 potential candidates (**Figure 4A**). Genes upregulated within the surfaceome included: *TSPAN11*, *CLDN10*, *SLC26A9*, *SLC7A2*, *TREM1*, *SLC46A2*, *PCDHB11*, *CHL1*, *SCN9A*, *PARM1*, *TMPRSS6*, and *KIT* (ordered from higher to lower fold increase) (**Supplementary Table 3**). The functions of the identified genes included regulation of cytokines signaling in immune cells (*KIT* and *TREM1*), import and transport of amino acids and molecules across plasma membrane (*SLC7A2, PARM1, KIT, SLC26A9*, and *SCN9A*), regulation of nucleotide-binding oligomerization domain (*SLC46A2*), regulation of action potential and ion homeostasis (*TMPRSS6*) and cell adhesion (*CLDN10* and *PCDHB11*) (**Figure 4B**). From these 12 genes, *CLDN10* gained our attention in the volcano plot due to its increased expression and significant p-value (**Figure 4C**).

### Expression of Surfaceome Candidates Associated With Prognosis

**Figure 5:**
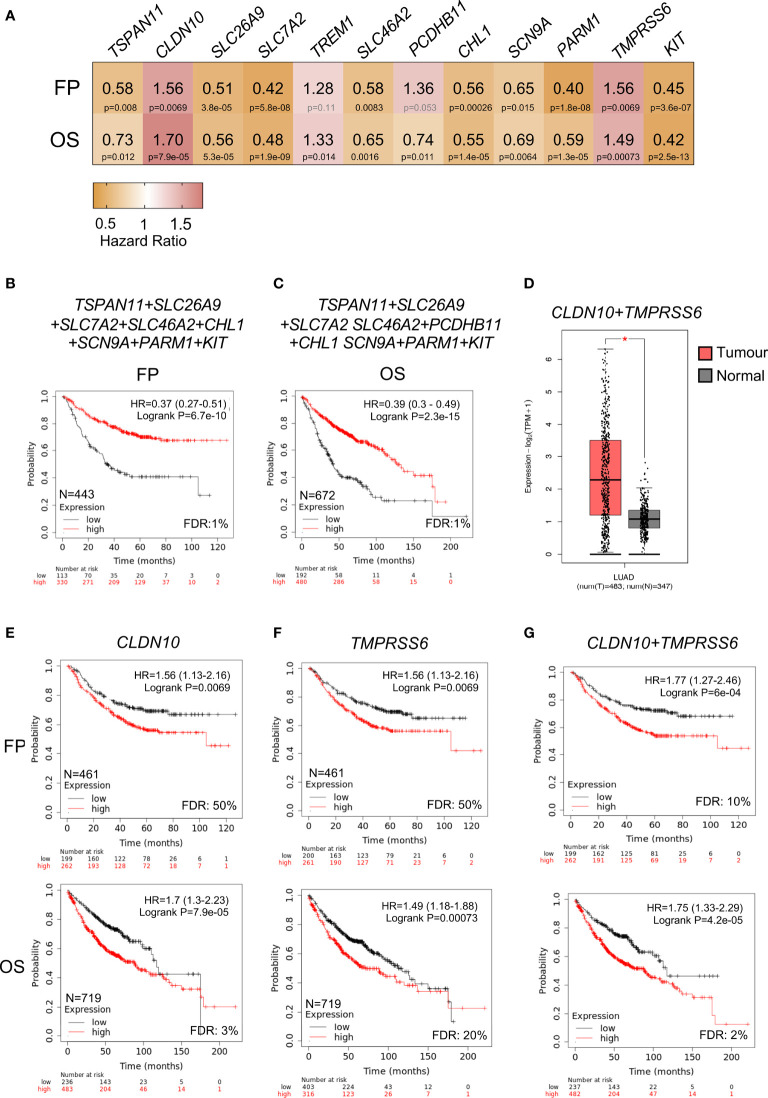
Correlation between expression of upregulated surfaceome genes and LUAD survival. **(A)** Heatmap showing the HR (yellow color indicates good outcome while red predicts poor prognosis) and p-values (black for significant and grey for no significant) of twelve selected genes. **(B)** Kaplan-Meier survival plots showing the association between combined expression of surfaceome signature with good prognosis for FP (*TSPAN11, SLC26A9, SLC7A2, SLC46A2, CHL1, SCN9A, PARM1*, and *KIT)* and in **(C)** for OS (*TSPAN11, SLC26A9, SLC7A2, SLC46A2, PCDHB11, CHL1, SCN9A, PARM1*, and *KIT*). **(D)** Combined expression of *CLDN10* and *TMPRSS6* genes in samples from LUAD (pink) or normal tissue (grey). Kaplan-Meier survival plots showing the association between the single (*CLDN10* in **E**; and *TMPRSS6* in **F**) or combined gene expression levels (*CLDN10* and *TMPRSS6* in **G**) with FP (above) and OS (below) for LUAD.

Among the previous identified genes, we next explored those that were associated with detrimental clinical prognosis. When studding those associated with FP and OS, we observed that only two genes *CLDN10* and *TMPRS6S* predicted for negative prognosis (**Figure 5A** and **Supplementary Table 4**). Pooling together the rest of the genes with good prognosis for FP (*TSPAN11, SLC26A9, SLC7A2, SLC46A2, CHL1, SCN9A, PARM1*, and *KIT*) and for OS (*TSPAN11, SLC26A9, SLC7A2, SLC46A2, PCDHB11, CHL1, SCN9A, PARM1*, and *KIT)*, we observed the best outcome for FP (HR:0.37, 99% CI 0.27–0.51, log rank p = 6.7 × 10^−10^) (**Figure 5B**) and OS (HR:0.39, 99% CI 0.3–0.49, log rank p = 2.3 × 10^−15^) (**Figure 5C**) supporting the notion that the function of *CLDN10* and *TMPRS6S* play an oncogenic role in this tumor subtype. Of note, the expression of both genes was clearly increased in LUAD tumors compared with normal tissues (**Figure 5D**). A higher expression of TMPRSS6 or CLDN10 individually could be detected in other tumors (**Supplementary Figures 4A, B** respectively), while a higher combined expression was observed in ovarian serous cystadenocarcinoma (OV), thyroid carcinoma (THCA), and uterine corpus endometrial carcinoma (UCEC) apart from LUAD (**Supplementary Figure 4C**).

The analysis of CLDN10 demonstrated a clear association with worse prognosis, FP (HR:1.56, 95% CI 1.13–2.16, log rank p = 0.0069); and OS (HR:1.7, 95% CI 1.3–2.23, log rank p = 7.9 × 10^−5^) (**Figure 5E**). A similar finding was observed with *TMPRSS6*, for FP (HR:1.56, 95% CI 1.13–2.16, log rank p = 0.0069) and OS, (HR:1.49, 95% CI 1.18–1.88, log rank p = 0.00073) (**Figure 5F**). The combined evaluation of both genes (*CLDN10* and *TMPRSS6*) demonstrated a stronger prediction, for FP: (HR:1.77, 95% CI 1.27–2.46, log rank p = 6 × 10^−4^), and OS: (HR:1.75, 95% CI 1.33–2.29, log ran k p = 4.2 × 10^−5^) (**Figure 5G**). Globally, these data demonstrate the association of these two genes with detrimental prognosis in NSCLC adenocarcinoma.

### *CLDN10* and *TMPRSS6* Expression in Lung Cancer and Normal Tissues

**Figure 6:**
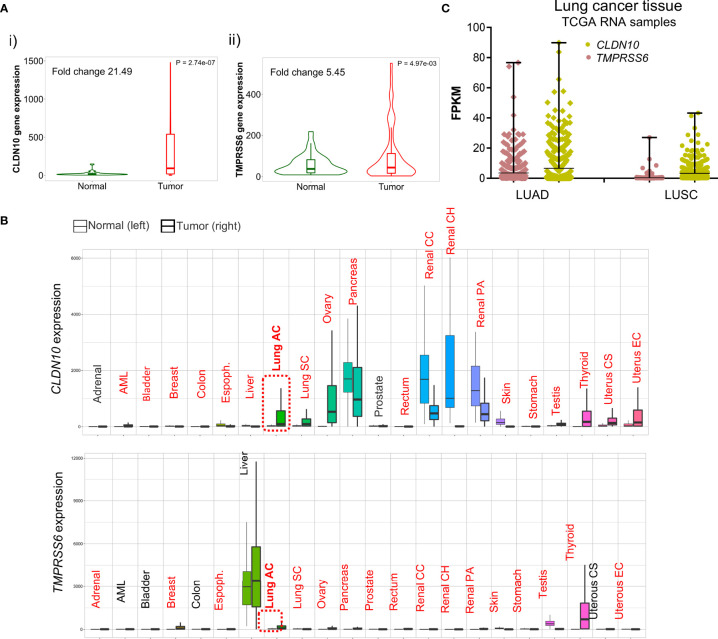
Differential expression of *CLDN10* and *TMPRSS6* between normal and tumor samples. **(A)** Violin plot of the expression of *CLDN10* **(i)** and *TMPRSS6* **(ii)** in LUAD tumor sample and adjacent normal tissue. **(B)** Pan-cancer analysis displays the expression of *CLDN10* and *TMPRSS6* genes across all tissues comparing tumoral and normal ones. Highlighted in red those tissues with statistical significance (p<0.01) differential expression by a Mann–Whitney U test. **(C)** Graph that illustrates the RNA of *CLDN10* (pistachio) and *TMPRSS6* (salmon) in fragments per kilo base per million mapped reads (FPKM) in NSCLC histological subtypes LUAD and LUSC.

We next studied the expression of these two genes in comparison with non-transformed tissue. As shown in **Figure 6A**, CLDN10 (i) and TMPRSS6 (ii) had higher expression levels in LUAD compared with normal tissues. Then, we wanted to check if the higher expression of these two surface candidates was increased in K-RAS p.G12C tumoral samples compared to wildtype ones. We first perform the analysis in NSLC cell lines and selected those ones with mutations at the *K-RAS* G12C gene (**Supplementary Figure 5A**). The data obtained indeed show a higher expression of *CLDN10* (**Supplementary Figure 5B**) and *TMPRSS6* (**Supplementary Figure 5C**) when *K-RAS* harbors this mutation. Similar results were obtained when analyzing the expression of *CLDN10* (**Supplementary Figure 5D**) and *TMPRSS6* (**Supplementary Figure 5E**) in LUAD tumor samples. When evaluating the expression levels in LUAD compared with different types of non-transformed tissues, a higher expression was observed, with some exceptions like pancreatic and kidney tissue for CLDN10, and testis tissue for TMPRSS6 (**Figure 6B**). Finally, we found that the expression of these two genes was higher in LUAD than in LUSC tumors (**Figure 6C**), which is in line with the higher frequency of *K-RAS G12C* mutations in LUAD. These data globally confirm the exclusive expression of these genes in this tumor type.

## Discussion

In the present article we identify novel genes upregulated in *K-RAS G12C* mutated NSCLCs that could be used as targets to vectorize compounds against these tumors, or as surrogates of immune activation, to select patients or explore combinations with novel immune therapies.

Although a high grade of clinical activity has been described with novel immune therapies, the identification of responsive tumors is still limited to a reduced number of patients whose tumors harbor some biomarkers like PD-L1 or contain certain genomic alterations (10).

With the identification of compounds against druggable molecular vulnerabilities, the association of these agents with novel immunotherapies is a strategy to pursuit. In this context, and given the fact that some combinations, – like those combining PD (L)1 antibodies with EGFR inhibitors (18) –, have shown negative results, we aimed to identify deregulated genes that were linked with outcome and, at the same time, associated with different sets of immune populations. We selected a set of genes that were modified in tumors with *K-RAS G12C* mutations, including the upregulated HOPX, PDED4 and CRLF1 genes, that were in addition linked with favorable prognosis in terms of FP and OS. These genes are involved in the development and regulation of the immune system, like HOPX which is required for the conversion of CD4(+) T cells into regulatory T cells (Tregs) (22); PDED4, that plays a predominant role in propagating various T cell functions (23); and CRLF1, that induces B-cell expansion (24) and CD4(+) T cells accumulation (25). In this context, patients harboring this gene signature are associated with a more favorable prognosis and with a clear upregulation of B and CD4+ T cells. Of note, no association was observed for other cell populations including CD8+ T cells, neutrophils, macrophages or dendritic cells. These data are in line with the global downregulation of immune cells observed in LUAD patients harboring mutations at the *K-RAS G12C* gene and correlates with the presence of downregulated transcripts and reduced levels of neutrophils, macrophages and CD8+ T cells identified in this study. Therefore, strategies aimed to boost T cell activation, innate response and antigen presentation could be pursuit in this cohort. In line with this idea, a recent article showed a synergistic interaction between the K-RAS G12C inhibitor adagrasib and PD (L)1 inhibitors that led to an outstanding antitumoral activity (19).

Resistance due to secondary mutations is the principal mechanism of failure to tyrosine kinase inhibitors. Recent data also suggest that resistance to *K-RAS G12C* inhibitors can rely on acquired *K-RAS* alterations including G12D/R/V/W, G13D, Q61H, R68S, H95D/Q/R, and Y96C mutations or high-level amplification of the *K-RAS^G12C^* allele (12). These alterations could be potentially overcome if the *K-RAS* oncogene is degraded with a PROTAC compound. Thus, identification of targets that are primarily, but not exclusively, expressed in this tumor type could limit the narrow therapeutic index expected when using novel PROTAC agents (13, 26).

Targeting pan-essential genes *via* agents with a narrow therapeutic index has shown to induce toxicity thereby limiting clinical development. This has been the case of agents targeting CDK9, among others (3). Therefore, guiding anticancer agents to facilitate the targeting of the right cell population is a main objective in drug development.

In our study, by interrogating TCGA NSCLC samples with *K-RAS G12C* mutated tumors, we identified 12 genes that were highly expressed and potentially present in the cellular membrane compared with normal tissues. We focused only on genes linked with detrimental prognosis: *CLDN10* and *TMPRSS6*. CLDN10 belongs to the claudin family which are integral membrane proteins and components of tight junction strands. They play and important role in cell polarity and signal transduction, and are present in the intercellular space between epithelial or endothelial components. There are different reports that establish a correlation between the expression of CLDN10 and survival, with some articles associating their increased levels with favourable prognosis (27, 28), and others with detrimental outcome (29). TMPRSS6 is a type II transmembrane serine proteinase involved in matrix remodelling. TMPRSS6 has an essential role in iron homeostasis and has been linked to iron-refractory iron deficiency anaemia (30, 31). However, its role in cancer is less understood, and there is very limited published data.

An interesting observation is that expression of neither CLDN10 nor TMPRSS6 were associated with any immune population (**Supplementary Figure 4D**), suggesting that they are present in the primary tumor and not in the stromal compartment. Another relevant observation is that the expression of these genes is higher in LUAD than in non-transformed tissues with the exception of pancreas and kidney for CLDN10 and TMPRSS6 in liver. Of note, TMPRSS6 is highly expressed in liver cancer. In addition, expression of both genes was high in LUAD compared with lung squamous carcinoma (LUSC) what aligns with the high frequency of *K-RAS G12C* mutation in LUAD.

We are aware that our study is an *in silico* analysis and that further evaluation in clinical samples from patients should be ideally performed. However, the application of well stablished bioinformatic tools together with the use of independent datasets limits the chance of having a false positive result.

In summary, in this work we described a set of immune-associated genes whose expression is deregulated in NSCLC harbouring *K-RAS G12C* mutations. The reduced presence of effector immune populations justifies the combination with effector T cell stimulators and agents that boost the antigen presentation effect. In addition, the reported signature linked with CD4+ T and B cells could be used to select patients with favourable outcome within this group of patients. Finally, we reported two genes that are upregulated in this particular tumor subtype and which could be used to deliver novel compounds.

## Materials and Methods

### Identification of *K-RAS* Mutations in Lung Cancer Patients, Data Collection and Processing

We used data contained at cBioportal (www.cbioportal.org) (accessed in July, 2021) (32, 33) to explore the alterations of *K-RAS* gene in patients with Non-Small Cell Lung Cancer, including Lung Squamous Cell Carcinoma (LUSC) and Lung Adenocarcinoma (LUAD). We used two different dataset per cancer type: Firehose Legacy (n=178) and TCGA, PanCancer Atlas (n=469) (34) for LUSC; and MSKCC, 2020 (n=604) and Firehose Legacy (n=230) for LUAD. This web resource also provides mutated variants mapped to genomic domains. Protein expression in cell membrane was identified using the Human Surfaceome Atlas (https://wlab.ethz.ch/surfaceome/) (accessed in June, 2021) (35).

Approved and Investigational drugs against *K-RAS* mutant variants in patients with NSCLC were identified using U.S. Food and Drug Administration website (https://www.fda.gov/), (last accessed on June, 2021) and Clinical Trials database (https://clinicaltrials.gov/), (accessed on June, 2021), respectively.

### Functional Annotation of De-Regulated Genes

We used the publicly available EnrichR online platform (https://maayanlab.cloud/Enrichr/) (accessed on July 2021) (36) to address the Gene Ontology Biological function related to each gene set. We grouped similar biological functions and represented them with the higher combined score of most relevant pathways.

### Outcome Analysis

The KM Plotter Online tool (37) (https://kmplot.com/analysis/, last accessed on July, 2021) was used to evaluate the relationship between up-regulated gene’s expression and clinical outcome in patients with Lung Adenocarcinoma. This open access database contains 3,452 lung cancer samples and allowed us to investigate Free Progression (FP) and Overall Survival (OS) of up-regulated genes in the Lung Adenocarcinoma subtype. False discovery rate (FDR) indicates replicable associations across multiple studies.

### Expression Analysis

The analysis comparing the expression level of individual genes and combined signature between normal lung tissue (*n* = 347) and Lung Adenocarcinoma samples (*n* = 483) was performed with GTEx and TCGA data using GEPIA2 web server (Gene Expression Profiling Interactive Analysis; http://gepia2.cancer-pku.cn/) (last accessed on June, 2021) (38).

Differential gene-expression analysis in tumor and normal tissues was performed using the web tool TNMplot (39) (https://tnmplot.com/analysis/, accessed on July, 2021).

The analysis comparing the expression level of individual genes in *K-RAS* G12C samples compared with the rest was done using data from the Cancer Dependency Map (DepMap) portal (https://depmap.org/portal/, accessed on October, 2021) for cell lines; and UCSC Xena portal (http://xena.ucsc.edu/, accessed on October, 2021) (40) for TCGA LUAD tumor samples.

### Correlation Between Gene Expression and Immune Cell Infiltration

To explore the associations between gene expression and immune infiltration cells we used TIMER2.0 (http://timer.cistrome.org/, accessed on July, 2021). TIMER provides 4 modules (Gene, Mutation, sCNA and Outcome) to explore the association between immune infiltrates and genomic changes (41). Gene-correlation module was used to link gene expression with activation of T cell markers.

### Graphical Design

Bars, heatmaps, dot plots and volcano plots were represented using GraphPad Prism software (GraphPad Software, San Diego, CA, USA) in terms of absolute counts, relative frequencies, and hazard ratios. Kaplan-Meier curves were produced by specific online tools as previously described (Kaplan-Meier plotter).

## Data Availability Statement

The results presented in this study are based upon data generated by the cBioPortal (www.cbioportal.org) and TCGA Research Network (https://www.cancer.gov/tcga). Raw data from our own analysis can be provided by asking directly to the corresponding authors of this manuscript.

## Ethics Statement

Ethical review and approval was not required for the study on human participants in accordance with the local legislation and institutional requirements. The patients/participants provided their written informed consent to participate in this study. 

## Author Contributions

AO conceived the study and did the original design of analysis. AA-S, ECM, and GF-H searched the data and performed the analyses. BG provided bioinformatic assistance in data search and analysis. GV, PP-S, and AP provided support in data interpretation. AO and ECM wrote the manuscript. All authors reviewed, included modification and approved the final version of the manuscript. All authors have read and agreed to the published version of the manuscript.

## Funding

AO’s lab is supported by the Instituto de Salud Carlos III (ISCIII, PI19/00808); CRIS Cancer Foundation, ACEPAIN, and Diputación de Albacete. AP’s lab is founded by the Ministry of Economy and Competitiveness of Spain (BFU2015-71371-R and FEDER). The CRIS Cancer Foundation supports both research groups. BG’s lab is supported by the 2018-2.1.17-TET-KR-00001, 2020-1.1.6-JÖVŐ-2021-00013, 2018-1.3.1-VKE-2018-00032 and 2020-4.1.1.-TKP2020 grants of the NKFIH, Hungary. EC is supported by a “Juan de la Cierva incorporación” contract of the Spanish Ministry of Science and Innovation with Ref. IJC2019-041728-I. AA-S is funded by a fellowship from the Junta de Comunidades de Castilla-La Mancha.

## Conflict of Interest

The authors declare that the research was conducted in the absence of any commercial or financial relationships that could be construed as a potential conflict of interest.

## Publisher’s Note

All claims expressed in this article are solely those of the authors and do not necessarily represent those of their affiliated organizations, or those of the publisher, the editors and the reviewers. Any product that may be evaluated in this article, or claim that may be made by its manufacturer, is not guaranteed or endorsed by the publisher.

## Supplementary Material

Supplementary Figure 1 Identification of *K-RAS* most common genomic alterations in patients with NSCLC by molecular subtypes. **(i)** Percentage of amplifications, mutations and deletions of *K-RAS* gene in patients with Squamous Cell Carcinoma or Adenocarcinoma. **(ii)** Graphical representation of the percentage of *K-RAS* genomic alterations according to TCGA, Firehose Legacy and MSKCC, 2020 databases in **(A)** or to TCGA, PanCancer Atlas and TCGA, Firehose Legacy databases in **(B)**.

Supplementary Figure 2 Negative correlation between the expression of upregulated genes (*CLRF1, HOPX, IRS2, KIT, PDE4D*, and *SMOC1*) and most of immune infiltrates (CD8+ T cells, Neutrophils, Macrophages and dendritic cells). A green square encircles the dots with higher expression and little infiltration level.

Supplementary Figure 3 Positive correlation between the expression of downregulated genes (*CD24, CDK6, HDAC9, TIAM1, TRFC, VTRC1, and VAV3*) and most of immune infiltrates (CD8+ T cells, Neutrophils, Macrophages and dendritic cells). A yellow square encircles the dots with less expression and more infiltration level.

Supplementary Figure 4 Expression of *CLDN10* and *TMPRSS6* in different human cancer types. Bar graph showing the expression of individual *CLDN1*0 (in **(A)**, *TMPRSS6* in **(B)** or both genes combined **(C)** in those cancer types where expression is significantly higher in tumor samples than in normal tissue **(D).** No correlation between *CLDN10* and *TMPRSS6* gene expression with most of immune infiltrates.

Supplementary Figure 5 Higher expression of *CLDN10* and *TMPRSS6* in *K-RAS* p.G12C. **(A)** Table presenting 15 NSLC cell lines that present the G12C variant in *K-RAS* gene. Bar graph showing the expression of *CLDN1*0 in **(B)**, and *TMPRSS6* in **(C)** in 92 NSLC cell lines compared to those 15 selected in A. Expression of *CLDN1*0 in **(D)**, and *TMPRSS6* in **(E)** in LUAD tumor samples comparing those that harbor or not the G12C mutation.

Supplementary Table 1 Investigational and approved drugs against *K-RAS* identified mutations. Specific K-RAS mutation, name of the drug, status (approved or investigational), identification code (NCT) and clinical studies with links and phases are included. Intervention is included if the drug is given in combination with others.

Supplementary Table 2 Gene functions of thirteen selected deregulated genes.

Supplementary Table 3 Upregulation details of cell surface-related genes analyzed. Name of the gene, mean of expression in mutant and wildtype K-RAS tumors, fold change (FC), direction and p-value are included.

Supplementary Table 4 Kaplan-Meier survival values of cell surface-related genes. Table includes the name of the gene, the hazard ratio (HR) (in blue, significant good prognosis, and in red, bad one), p-value and fold discovery rate (FDR) for FP and in LUAD patients.
