# Supplementary material for: Transcriptomic Mapping of Non-Small Cell Lung Cancer K-RAS p.G12C Mutated Tumors: Identification of Surfaceome Targets and Immunologic Correlates
Source: Front Immunol. 2022 Feb 1;12:786069. doi: 10.3389/fimmu.2021.786069 (PMC8843839; doi:10.3389/fimmu.2021.786069)

Supplementary Figure 1

A

i)

Non-Small Cell Lung Cancer		
Lung Squamous Cell Carcinoma (TCGA, Firehose Legacy)		
178 patients/samples		
Amplification	Mutation	Multiple alterations
4.49% (8 cases)	1.12% (2 cases)	0
Lung Adenocarcinoma (MSKCC, 2020)		
604 patients/samples		
Amplification	Mutation	Multiple alterations
0.33% (2 cases)	37.91% (229 cases)	0.17% (1 case)

ii)

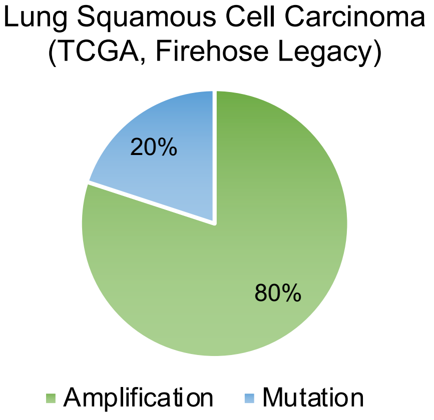

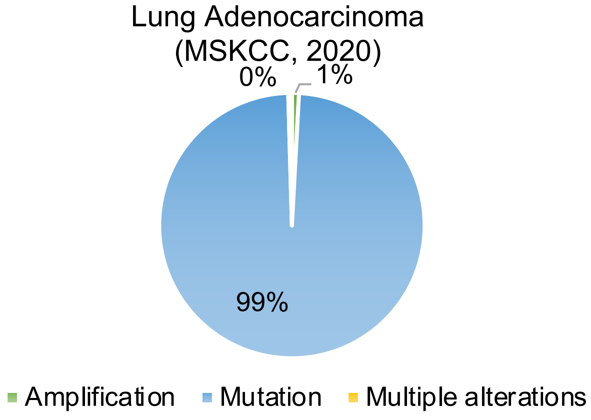

B

i)

Non-Small Cell Lung Cancer			
Lung Squamous Cell Carcinoma (TCGA, PanCancer Atlas)			
469 patients/samples			
Amplification	Mutation	Multiple alterations	
3.2% (15 cases)	1.49% (7 cases)	0	
Lung Adenocarcinoma (TCGA, Firehose Legacy)			
230 patients/samples			
Amplification	Mutation	Deep deletion	Multiple alterations
2.61% (6 cases)	29.13% (67 cases)	0.43% (1 case)	3.48% (8 cases)

ii)

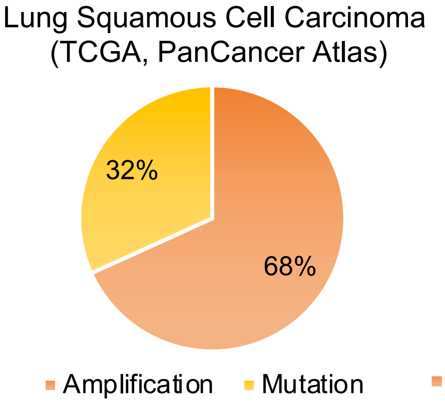

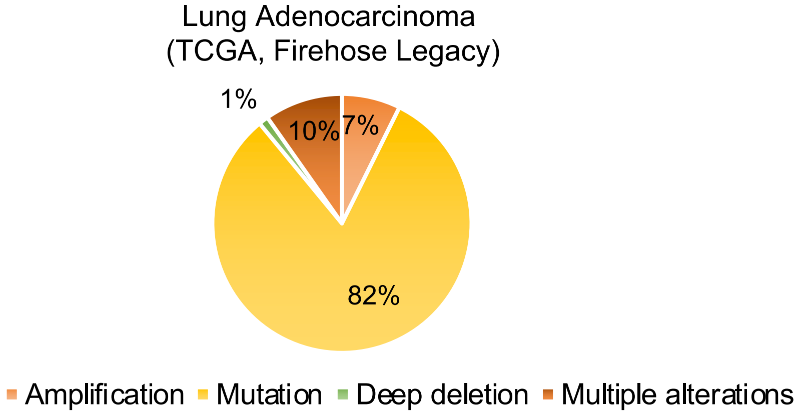

Supplementary Figure 2

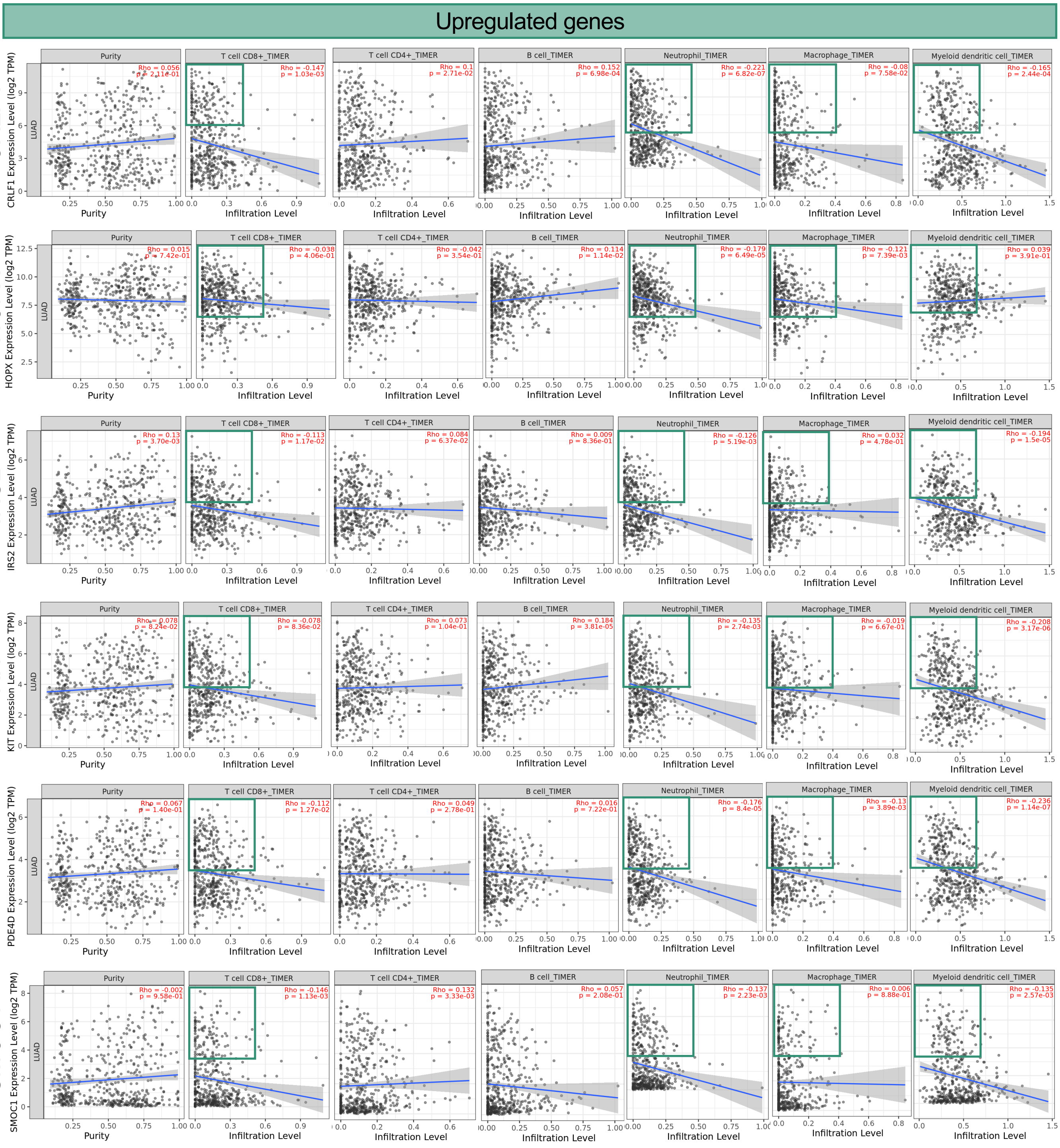

Supplementary Figure 3

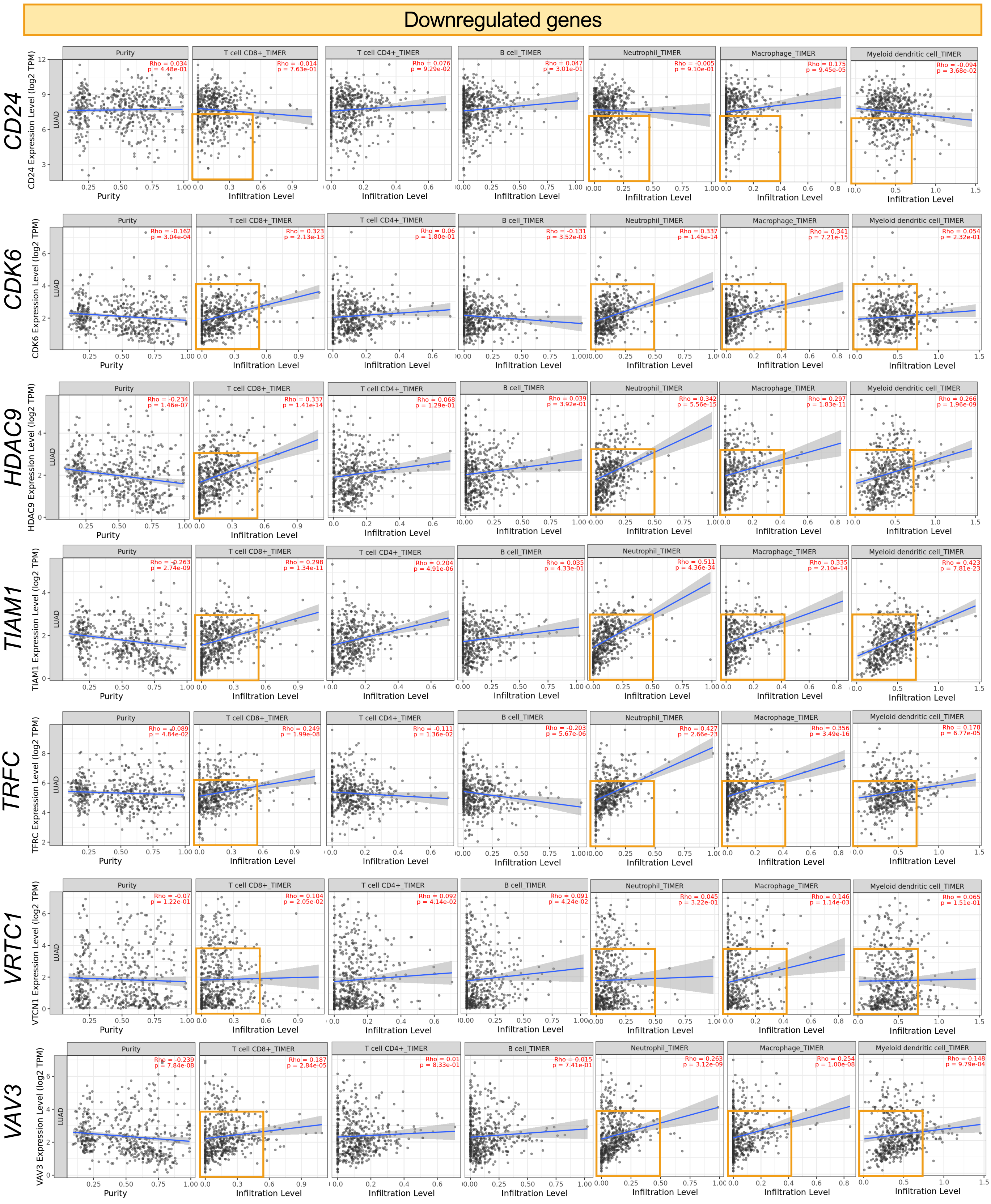

Supplementary Figure 4

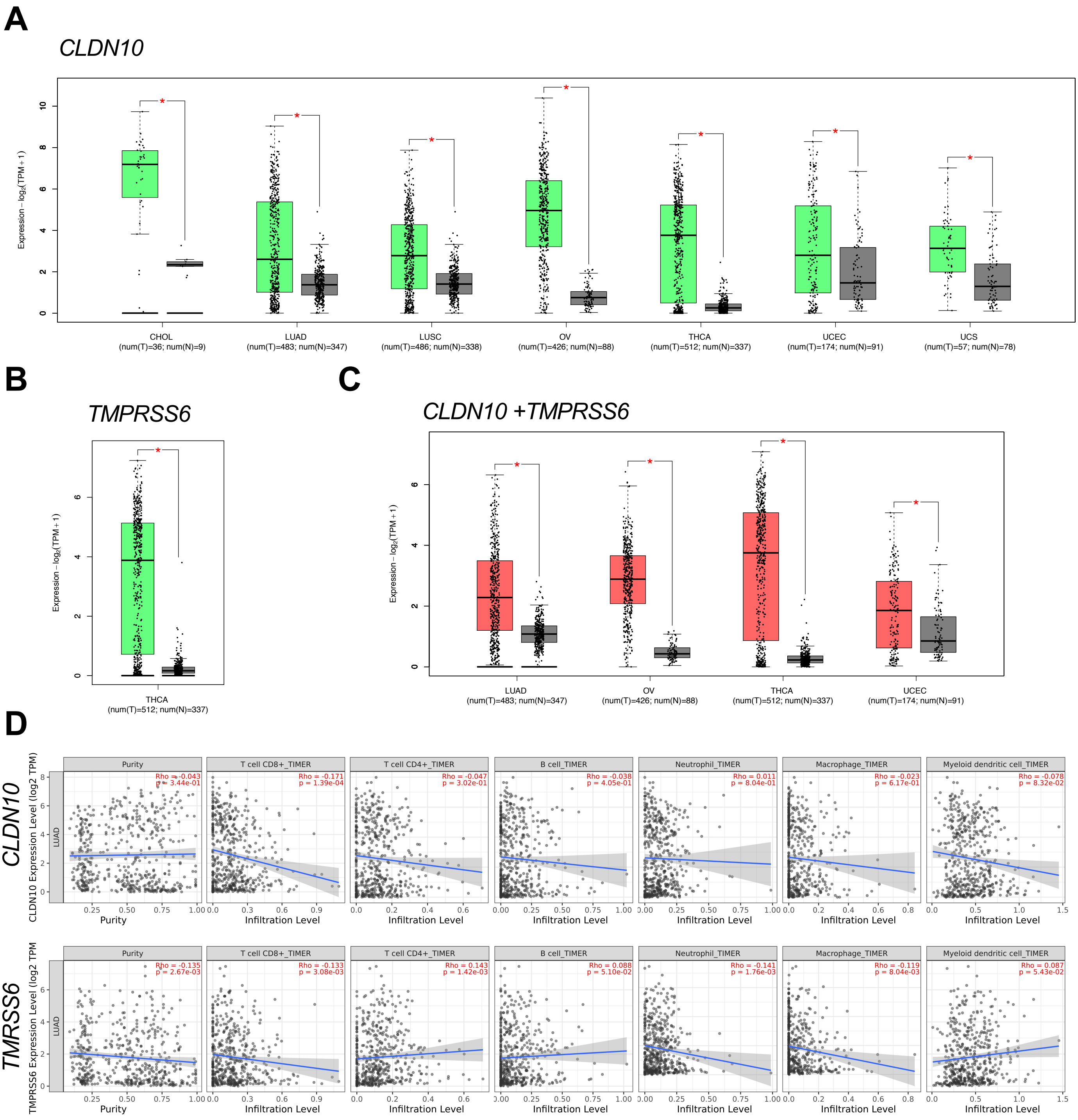

Supplementary Figure 5

A

**NSLC cell lines with *K-RAS* p.G12C**	
NCIH1373	HCC1171
HOP62	SW1573
NCIH358	IALM
NCIH2122	CALU1
NCIH1792	NCIH1385
NCIH2030	HCC44
LU99	LU65
NCIH23	

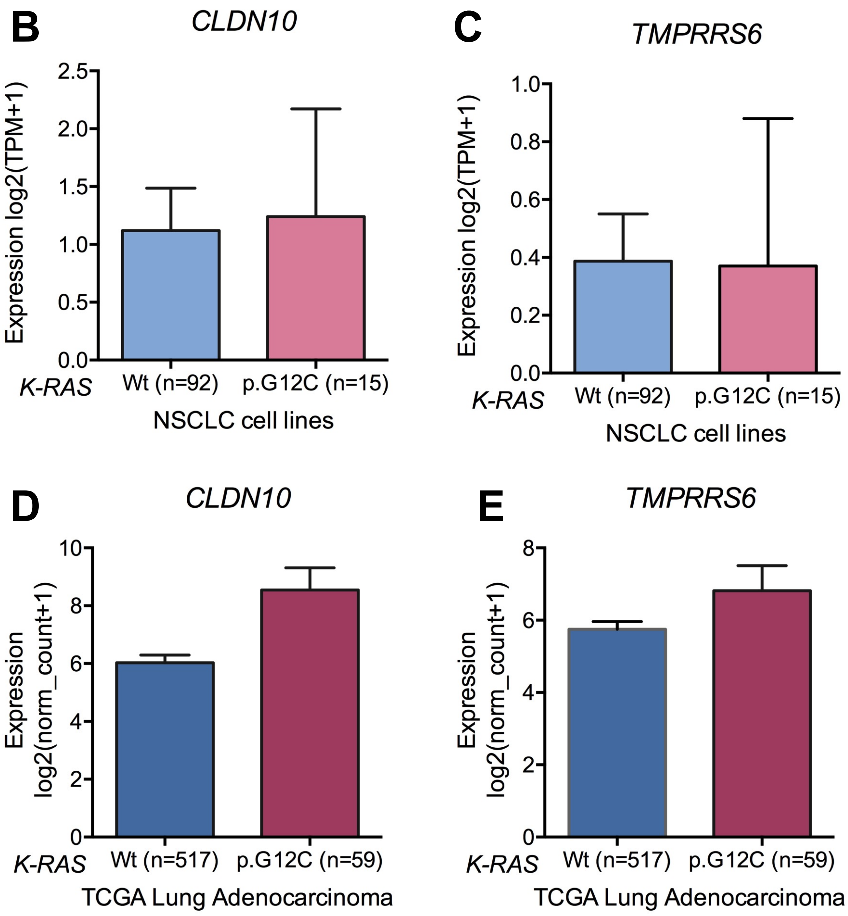

Supplement: Supplementary Figure 1 — Identification of K-RAS most common genomic alterations in patients with NSCLC by molecular subtypes. (i) Percentage of amplifications, mutations and deletions of K-RAS gene in patients with Squamous Cell Carcinoma or Adenocarcinoma. (ii) Graphical representation of the percentage of K-RAS genomic alterations according to TCGA, Firehose Legacy and MSKCC, 2020 databases in (A) or to TCGA, PanCancer Atlas and TCGA, Firehose Legacy databases in (B). [file DataSheet_1.pdf]
