# Supplementary material for: Transcriptomic Mapping of Non-Small Cell Lung Cancer K-RAS p.G12C Mutated Tumors: Identification of Surfaceome Targets and Immunologic Correlates
Source: Front Immunol. 2022 Feb 1;12:786069. doi: 10.3389/fimmu.2021.786069 (PMC8843839; doi:10.3389/fimmu.2021.786069)
Supplement: Supplementary Table 1 — Investigational and approved drugs against K-RAS identified mutations. Specific K-RAS mutation, name of the drug, status (approved or investigational), identification code (NCT) and clinical studies with links and phases are included. Intervention is included if the drug is given in combination with others. [file DataSheet_2.pdf]

Supplementary Table 1

KRAS mutation	Drug	Drug status	NCT	Clinical trial	Link	Intervention	Ph
**G12C**	AMG 510	Approved	NCT04625647	Testing the Use of Targeted Treatment (AMG 510) for KRAS G12C Mutated Advanced Non-squamous Non-small Cell Lung Cancer (A Lung-MAP Treatment Trial)	https://clinicaltrials.gov/ct2/show/NCT04625647	Sotorasib	2
			NCT04303780	Study to Compare AMG 510 "Proposed INN Sotorasib" With Docetaxel in Non Small Cell Lung Cancer (NSCLC) (CodeBreak 200).	https://clinicaltrials.gov/ct2/show/NCT04303780	Docetaxel	3
			NCT04667234	Expanded Access of AMG 510 (Sotorasib)	https://www.clinicaltrials.gov/ct2/show/NCT04667234		
	MRTX849	Investigational	NCT04685135	Phase 3 Study of MRTX849 vs Docetaxel in Patients With Advanced Non-Small Cell Lung Cancer With KRAS G12C Mutation (KRYSTAL-12)	https://clinicaltrials.gov/ct2/show/NCT04685135	Docetaxel	3
			NCT04613596	Phase 2 Trial of MRTX849 Plus Pembrolizumab for NSCLC With KRAS G12C Mutation KRYSTAL-7	https://clinicaltrials.gov/ct2/show/NCT04613596	Pembrolizumab	2
			NCT04330664	Phase 1/2 Study in Patients With Cancer Having a KRAS G12C Mutation KRYSTAL 2	https://clinicaltrials.gov/ct2/show/NCT04330664	TNO155	2
	JNJ-74699157	Investigational	NCT04006301	First-in-Human Study of JNJ-74699157 in Participants With Tumors Harboring the KRAS G12C Mutation	https://clinicaltrials.gov/ct2/show/NCT04006301		1
	LY3499446	Investigational	NCT04165031	A Study of LY3499446 in Participants With Advanced Solid Tumors With KRAS G12C Mutation	https://clinicaltrials.gov/ct2/show/NCT04165031	Abemaciclib<br>Cetuximab<br>Erlotinib<br>Docetaxel	2
	GDC-6036	Investigational	NCT04449874	A Study to Evaluate the Safety. Pharmacokinetics. and Activity of GDC-6036 Alone or in Combination in Participants With Advanced or Metastatic Solid Tumors With a KRAS G12C Mutation	https://clinicaltrials.gov/ct2/show/NCT04449874	Atezolizumab<br>Cetuximab<br>Bevacizumab<br>Erlotinib	1
	JDQ443	Investigational	NCT04699188	Study of JDQ443 in Patients With Advanced Solid Tumors Harboring the KRAS G12C Mutation	https://clinicaltrials.gov/ct2/show/NCT04699188	TNO155<br>Spartalizumab	2
	Cisplatin-Pemetrexed vs Carboplatin-Paclitaxel-Bevacizumab	Approved	NCT02743923	Cisplatin-Pemetrexed Compared With Carboplatin-Paclitaxel-Bevacizumab in KRAS Mutated Non-small Cell Lung Cancer	https://clinicaltrials.gov/ct2/show/NCT02743923	Carboplatin<br>Paclitaxel<br>Bevacizumab<br>Pemetrexed<br>Cisplatin	3
	V941	Investigational	NCT03948763	A Study of mRNA-5671/V941 as Monotherapy and in Combination With Pembrolizumab (V941-001)	https://clinicaltrials.gov/ct2/show/NCT03948763	Pembrolizumab	1
**G12D**	Bortezomib	Approved	NCT01833143	Bortezomib in KRAS-Mutant Non-Small Cell Lung Cancer in Never Smokers or Those With KRAS G12D	https://clinicaltrials.gov/ct2/show/NCT01833143	Acyclovir	2
	ELI-002	Investigational	NCT04853017	A Study of ELI-002 in Subjects With KRAS Mutated Pancreatic Ductal Adenocarcinoma (PDAC) and Other Solid Tumor (AMPLIFY-201)	https://clinicaltrials.gov/ct2/show/NCT04853017		2
	V941	Investigational	NCT03948763	A Study of mRNA-5671/V941 as Monotherapy and in Combination With Pembrolizumab (V941-001)	https://clinicaltrials.gov/ct2/show/NCT03948763	Pembrolizumab	1
	BCA101	Investigational	NCT04429542	Study of Safety and Tolerability of BCA101 Alone and in Combination With Pembrolizumab in Patients With EGFR-driven Advanced Solid Tumors	https://clinicaltrials.gov/ct2/show/NCT04429542	Pembrolizumab	1
**G12V**	VS-6766 v. VS-6766 + Defactinib	Investigational	NCT04620330	A Study of VS-6766 v. VS-6766 + Defactinib in Recurrent G12V or Other KRAS-Mutant Non-Small Cell Lung Cancer	https://www.clinicaltrials.gov/ct2/show/NCT04620330	Defactinib	2
	Cisplatin-Pemetrexed vs Carboplatin-Paclitaxel-Bevacizumab	Approved	NCT02743923	Cisplatin-Pemetrexed Compared With Carboplatin-Paclitaxel-Bevacizumab in KRAS Mutated Non-small Cell Lung Cancer	https://clinicaltrials.gov/ct2/show/NCT02743923	Carboplatin<br>Paclitaxel<br>Bevacizumab<br>Pemetrexed<br>cisplatin	3
	V941	Investigational	NCT03948763	A Study of mRNA-5671/V941 as Monotherapy and in Combination With Pembrolizumab (V941-001)	https://clinicaltrials.gov/ct2/show/NCT03948763	Pembrolizumab	1
**G13D**	BCA101	Investigational	NCT04429542	Study of Safety and Tolerability of BCA101 Alone and in Combination With Pembrolizumab in Patients With EGFR-driven Advanced Solid Tumors	https://clinicaltrials.gov/ct2/show/NCT04429542	Pembrolizumab	1
	V941	Investigational	NCT03948763	A Study of mRNA-5671/V941 as Monotherapy and in Combination With Pembrolizumab (V941-001)	https://clinicaltrials.gov/ct2/show/NCT03948763	Pembrolizumab	1
**G12R**	ELI-002	Investigational	NCT04853017	A Study of ELI-002 in Subjects With KRAS Mutated Pancreatic Ductal Adenocarcinoma (PDAC) and Other Solid Tumor (AMPLIFY-201)	https://clinicaltrials.gov/ct2/show/NCT04853017		2

Supplementary Table 2

Downregulated Genes

- *VAV3*

Exchange factor for GTP-binding proteins RhoA. RhoG and. to a lesser extent. Rac1. Binds physically to the nucleotide-free states of those GTPases. Plays an important role in angiogenesis. Its recruitment by phosphorylated EPHA2 is critical for EFNA1-induced RAC1 GTPase activation and vascular endothelial cell migration and assembly.

- *TFRC*

Cellular uptake of iron occurs via receptor-mediated endocytosis of ligand-occupied transferrin receptor into specialized endosomes. Endosomal acidification leads to iron release. The apotransferrin-receptor complex is then recycled to the cell surface with a return to neutral pH and the concomitant loss of affinity of apotransferrin for its receptor. Positively regulates T and B cell proliferation through iron uptake. Acts as a lipid sensor that regulates mitochondrial fusion by regulating activation of the JNK pathway

- *TIAM1*

This gene encodes a RAC1-specific guanine nucleotide exchange factor (GEF). GEFs mediate the exchange of guanosine diphosphate (GDP) for guanosine triphosphate (GTP). The binding of GTP induces a conformational change in RAC1 that allows downstream effectors to bind and transduce a signal. This gene thus regulates RAC1 signaling pathways that affect cell shape. migration. adhesion. growth. survival. and polarity. as well as influencing actin cytoskeletal formation. endocytosis. and membrane trafficking. This gene thus plays an important role in cell invasion. metastasis. and carcinogenesis. In addition to RAC1. the encoded protein activates additional Rho-like GTPases such as CDC42. RAC2. RAC3 and RHOA.

- *CDK6*

The protein encoded by this gene is a member of the CMGC family of serine/threonine protein kinases. This kinase is a catalytic subunit of the protein kinase complex that is important for cell cycle G1 phase progression and G1/S transition. The activity of this kinase first appears in mid-G1 phase. which is controlled by the regulatory subunits including D-type cyclins and members of INK4 family of CDK inhibitors. This kinase. as well as CDK4. has been shown to phosphorylate. and thus regulate the activity of. tumor suppressor protein Rb

- *CD24*

This gene encodes a sialoglycoprotein that is expressed on mature granulocytes and B cells and modulates growth and differentiation signals to these cells. The precursor protein is cleaved to a short 32 amino acid mature peptide which is anchored via a glycosyl phosphatidylinositol (GPI) link to the cell surface.

- *HDAC9*

Responsible for the deacetylation of lysine residues on the N-terminal part of the core histones (H2A. H2B. H3 and H4). Histone deacetylation gives a tag for epigenetic repression and plays an important role in transcriptional regulation. cell cycle progression and developmental events. Isoform 3 lacks active site residues and therefore is catalytically inactive. Represses MEF2-dependent transcription by recruiting HDAC1 and/or HDAC3.

- *VTCN1*

Negatively regulates T-cell-mediated immune response by inhibiting T-cell activation. proliferation. cytokine production and development of cytotoxicity. When expressed on the cell surface of tumor macrophages. plays an important role. together with regulatory T-cells (Treg). in the suppression of tumor-associated antigen-specific T-cell immunity. Involved in promoting epithelial cell transformation.

Upregulated Genes

- *IRS2*

Encodes the insulin receptor substrate 2. a cytoplasmic signaling molecule that mediates effects of insulin. insulin-like growth factor 1. and other cytokines by acting as a molecular adaptor between diverse receptor tyrosine kinases and downstream effectors. The product of this gene is phosphorylated by the insulin receptor tyrosine kinase upon receptor stimulation. as well as by an interleukin 4 receptor-associated kinase in response to IL4 treatment

- *SMOC1*

Plays essential roles in both eye and limb development. Probable regulator of osteoblast differentiation.

- *HOPX*

Atypical homeodomain protein which does not bind DNA and is required to modulate cardiac growth and development. Acts via its interaction with SRF. thereby modulating the expression of SRF-dependent cardiac-specific genes and cardiac development. Prevents SRF-dependent transcription either by inhibiting SRF binding to DNA or by recruiting histone deacetylase (HDAC) proteins that prevent transcription by SRF. May act as a tumor suppressor. Acts as a co-chaperone for HSPA1A and HSPA1B chaperone proteins and assists in chaperone-mediated protein refolding

- *KIT*

This gene was initially identified as a homolog of the feline sarcoma viral oncogene v-kit and is often referred to as proto-oncogene c-Kit. The canonical form of this glycosylated transmembrane protein has an N-terminal extracellular region with five immunoglobulin-like domains. a transmembrane region. and an intracellular tyrosine kinase domain at the C-terminus. Upon activation by its cytokine ligand. stem cell factor (SCF). this protein phosphorylates multiple intracellular proteins that play a role in the proliferation. differentiation. migration and apoptosis of many cell types and thereby plays an important role in hematopoiesis. stem cell maintenance. gametogenesis. melanogenesis. and in mast cell development. migration and function.

- *PDE4D*

The encoded protein has 3'.5'-cyclic-AMP phosphodiesterase activity and degrades cAMP. which acts as a signal transduction molecule in multiple cell types.

- *CRLF1*

In complex with CLCF1. forms a heterodimeric neurotropic cytokine that plays a crucial role during neuronal development (Probable). May also play a regulatory role in the immune system.

Supplementary Table 3

SURFACEOME PROTEINS					
Gene	Mean expression in *K-RAS* mutant tumours (n=57)	Mean expression in *K-RAS* wildtype tumours (n=451)	FC	Direction	p-value
TSPAN11	1812	763.14	2.37	upregulated	0.00041
CLDN10	1185.4	508.48	2.33	upregulated	9.75E-08
SLC26A9	3021.7	1446.85	2.09	upregulated	0.00984
SLC7A2	5828.09	3016.17	1.93	upregulated	0.00845
TREM1	2937.05	1608.93	1.83	upregulated	0.00899
SLC46A2	258.33	153.47	1.68	upregulated	0.000296
PCDHB11	229.33	136.59	1.68	upregulated	0.00107
CHL1	1565.28	942.56	1.66	upregulated	0.000161
SCN9A	549.39	340.24	1.61	upregulated	0.00507
PARM1	8012.47	5010.32	1.6	upregulated	0.000791
TMPRSS6	560.28	361.42	1.55	upregulated	0.0066
KIT	2971.02	1956.93	1.52	upregulated	0.00262

Supplementary Table 4

Gene	Kaplan-Meier Plotter Lung Adenocarcinoma					
	FP (N=443)			OS (N=672)		
	Hazard Ratio	p-value	FDR	Hazard Ratio	p-value	FDR
TSPAN11	0.58(0.38-0.87)	0.008	50%	0.73(0.57-0.93)	0.012	50%
CLDN10	1.56(1.13-2.16)	0.0069	50%	1.7(1.3-2.23)	7.9e-05	3%
SLC26A9	0.51(0.37 – 0.71)	3.8e-05	1%	0.56(0.42 – 0.74)	5.3e-05	2%
SLC7A2	0.42(0.3-0.58)	5.8e-08	1%	0.48(0.38-0.61)	1.9e-09	1%
TREM1	1.28(0.94-1.75)	0.11	100%	1.33(1.06-1.68)	0.014	50%
SLC46A2	0.58(0.38 – 0.87)	0.0083	50%	0.65(0.5 – 0.85)	0.0016	50%
PCDHB11	1.36(0.99-1.86)	0.053	100%	0.74(0.58-0.93)	0.011	50%
CHL1	0.56(0.41-0.77)	0.00026	5%	0.55(0.42-0.72)	1.4e-05	1%
SCN9A	0.65(0.45-0.92)	0.015	50%	0.69(0.53-0.9)	0.0064	50%
PARM1	0.4(0.28 – 0.55)	1.8e-08	1%	0.59(0.46-0.75)	1.3e-05	1%
TMPRSS6	1.56(1.13-2.16)	0.0069	50%	1.49(1.18-1.88)	0.00073	20%
KIT	0.45(0.33-0.62)	3.6e-07	1%	0.42(0.33-0.53)	2.5e-13	1%
CLDN10+TMPRSS6	1.77(1.27-2.46)	6E-04	10%	1.75(1.33-2.29)	4.2e-05	2%
